# Supplementary material for: Metabolomic profiling of rare cell populations isolated by flow cytometry from tissues
Source: eLife. 2021 Jan 20;10:e61980. doi: 10.7554/eLife.61980 (PMC7847306; doi:10.7554/eLife.61980)
Supplement: Transparent reporting form [file elife-61980-transrepform.docx]

***eLife’s* transparent reporting form**

We encourage authors to provide detailed information *within their submission* to facilitate the interpretation and replication of experiments. Authors can upload supporting documentation to indicate the use of appropriate reporting guidelines for health-related research (see [EQUATOR Network](http://www.equator-network.org/%20)), life science research (see the [BioSharing Information Resource](https://biosharing.org/)), or the [ARRIVE guidelines](http://www.plosbiology.org/article/info:doi/10.1371/journal.pbio.1000412) for reporting work involving animal research. Where applicable, authors should refer to any relevant reporting standards documents in this form.

If you have any questions, please consult our Journal Policies and/or contact us: [editorial@elifesciences.org](mailto:editorial@elifesciences.org).

**Sample-size estimation**

- You should state whether an appropriate sample size was computed when the study was being designed
- You should state the statistical method of sample size computation and any required assumptions
- If no explicit power analysis was used, you should describe how you decided what sample (replicate) size (number) to use

Please outline where this information can be found within the submission (e.g., sections or figure legends), or explain why this information doesn’t apply to your submission:

Samples sizes were not pre-determined based on statistical power calculations but were based on our experience with these assays. This statement is in the methods section under “Assessing statistical significance”.

**Replicates**

- You should report how often each experiment was performed
- You should include a definition of biological versus technical replication
- The data obtained should be provided and sufficient information should be provided to indicate the number of independent biological and/or technical replicates
- If you encountered any outliers, you should describe how these were handled
- Criteria for exclusion/inclusion of data should be clearly stated
- High-throughput sequence data should be uploaded before submission, with a private link for reviewers provided (these are available from both GEO and ArrayExpress)

Please outline where this information can be found within the submission (e.g., sections or figure legends), or explain why this information doesn’t apply to your submission:

Numbers of biological replicates and numbers of independent experiments can be found in the figure legends. Where applicable, technical replicates were averaged and the averaged sample was considered one biological replicate. No data were excluded; however, mice sometimes died during experiments, presumably due to complications associated with irradiation and bone marrow transplantation. In those instances, data that had already been collected from the mice in interim analyses were included (such as donor contribution to peripheral blood chimerism over time).

**Statistical reporting**

- Statistical analysis methods should be described and justified
- Raw data should be presented in figures whenever informative to do so (typically when N per group is less than 10)
- For each experiment, you should identify the statistical tests used, exact values of N, definitions of center, methods of multiple test correction, and dispersion and precision measures (e.g., mean, median, SD, SEM, confidence intervals; and, for the major substantive results, a measure of effect size (e.g., Pearson's r, Cohen's d)
- Report exact p-values wherever possible alongside the summary statistics and 95% confidence intervals. These should be reported for all key questions and not only when the p-value is less than 0.05.

Please outline where this information can be found within the submission (e.g., sections or figure legends), or explain why this information doesn’t apply to your submission:

Raw data are shown as dot plots when N<15 per group. Summary statistics and p-values for each panel can be found in the figure. Statistical test and multiple comparisons methods are in figure legends, and detailed information as well as confidence intervals are available in the source data. Justification about statistical tests are described in the methods section under “Assessing statistical significance”.

(For large datasets, or papers with a very large number of statistical tests, you may upload a single table file with tests, Ns, etc., with reference to sections in the manuscript.)

**Group allocation**

- Indicate how samples were allocated into experimental groups (in the case of clinical studies, please specify allocation to treatment method); if randomization was used, please also state if restricted randomization was applied
- Indicate if masking was used during group allocation, data collection and/or data analysis

Please outline where this information can be found within the submission (e.g., sections or figure legends), or explain why this information doesn’t apply to your submission:

No formal randomization techniques were used. However, samples and mice were allocated randomly to experiments and processed in an arbitrary order. For analysis of LC-MS data from metabolomics experiments, A.W.D. provided LC-MS data to T.P.M and M.S.M.S in a manner that de-identified the samples. Following LC-MS data analysis, the samples were re-identified.

**Additional data files (“source data”)**

- We encourage you to upload relevant additional data files, such as numerical data that are represented as a graph in a figure, or as a summary table
- Where provided, these should be in the most useful format, and they can be uploaded as “Source data” files linked to a main figure or table
- Include model definition files including the full list of parameters used
- Include code used for data analysis (e.g., R, MatLab)
- Avoid stating that data files are “available upon request”

Please indicate the figures or tables for which source data files have been provided:

Source data files have been provided for each figure and supplementary figure. Source code for our metabolomics data analysis tool is hosted on a publicly accessible webpage, listed in the methods section under “Statistical analysis of metabolomics data”. All raw data have been provided for the AML versus ALL metabolite comparison experiments in the Figure 1 – supplementary table 4 source data file.
